# Supplementary material for: An Emerging Cross-Species Marker for Organismal Health: Tryptophan-Kynurenine Pathway
Source: Int J Mol Sci. 2022 Jun 4;23(11):6300. doi: 10.3390/ijms23116300 (PMC9181223; doi:10.3390/ijms23116300)
Supplement: Supplementary file 1 [file ijms-23-06300-s001.zip › ijms-1637473-supplementary.pdf]

Table S1. Clinical Implication of Kynurenine Metabolites.

Organ	Clinical Implication	Disease Phenotype	Reference(s)
**Brain**	Cancer	Cancer	[1]
	Neurobehaviour	Addiction	[2]
		Bipolar	[3], [4],[5], [6]
		Depression	[5], [7], [8], [9]
		Schizophrenia	[6], [5], [10]
	Neurodegenerative	ALS/MS	[11], [12]
		Alzheimer's Disease	[13], [14]
		Dementia	[15]
		Parkinson's Disease	[16], [17]
		REVIEW	[18], [19]
	Neurological Illness	CNS and PNS disorders	[20]
		Migraine	[21], [22]
		Neurotransmission	[23]
		REVIEW	[24]
		Somatic Symptoms	[25]
	Neuropathic Pain	Neuropathic Pain	[26], [27], [28]
**Breast**	Cancer	Cancer	[29]
**Heart**	Cardiometabolic	Cardiometabolic	[30]
		Cardiovascular Disease	[31],[32], [33], [34]
**Endocrine**	Metabolism	Obesity	[35], [36]
**Gastrointestinal**	Gastrointestinal Regulation	Gastrointestinal Disorders	[37]
		Gut Homeostasis	[38], [39]
		Inflammatory Bowel Disease	[40]
**Immune System**	Immunity	Endocrinopathies	[41]
		Inflammation	[42], [43]
		SARS-CoV-2	[44], [45]
**Kidney**	Kidney Disease		[46], [47]
**Liver**	Cancer	Cancer	[48], [49]
	Metabolism	Energy Homeostasis	[50], [51]
		Inflammation	[52]
		Liver Cirrhosis	[53]
		Liver Fibrosis	[54]
**MuscoSkeletal**	MuscoSkeletal	Inflammation	[55]
		SARS-CoV-2	[56]
**Placenta**	Reproduction	Pregnancy	[57], [58], [59], [60]
	Reproductive Disorders	Preeclampsia	[61], [62]
**REVIEW**	Cancer		[63]
	Gerontology		[64]
	All disease phenotypes		[65], [66], [67]

References

1. Sordillo, P.P.; Sordillo, L.A.; Helson, L. The Kynurenine Pathway: A Primary Resistance Mechanism in Patients with Glioblastoma. *Anticancer Res.* **2017**, *37*, 2159–2171. https://doi.org/10.21873/anticanres.11551.
2. Morales-Puerto, N.; Giménez-Gómez, P.; Pérez-Hernández, M.; Abuin-Martínez, C.; Gil de Biedma-Elduayen, L.; Vidal, R.; Gutiérrez-López, M.D.; O'Shea, E.; Colado, M.I. Addiction and the Kynurenine

Pathway: A New Dancing Couple? *Pharmacol. Ther.* **2021**, *223*, 107807. https://doi.org/10.1016/j.pharmthera.2021.107807.
3. Zhang, P.; Huang, H.; Gao, X.; Jiang, J.; Xi, C.; Wu, L.; Fu, Y.; Lai, J.; Hu, S. Involvement of Kynurenine Metabolism in Bipolar Disorder: An Updated Review. *Front. Psychiatry* **2021**, *12*, 677039. https://doi.org/10.3389/fpsyt.2021.677039.
4. Bartoli, F.; Misiak, B.; Callovini, T.; Cavaleri, D.; Cioni, R.M.; Crocamo, C.; Savitz, J.B.; Carrà, G. The Kynurenine Pathway in Bipolar Disorder: A Meta-Analysis on the Peripheral Blood Levels of Tryptophan and Related Metabolites. *Mol. Psychiatry* **2021**, *26*, 3419–3429. https://doi.org/10.1038/s41380-020-00913-1.
5. Marx, W.; McGuinness, A.J.; Rocks, T.; Ruusunen, A.; Cleminson, J.; Walker, A.J.; Gomes-da-Costa, S.; Lane, M.; Sanches, M.; Diaz, A.P.; et al. The Kynurenine Pathway in Major Depressive Disorder, Bipolar Disorder, and Schizophrenia: A Meta-Analysis of 101 Studies. *Mol. Psychiatry* **2021**, *26*, 4158–4178. https://doi.org/10.1038/s41380-020-00951-9.
6. Erhardt, S.; Schwieler, L.; Imbeault, S.; Engberg, G. The Kynurenine Pathway in Schizophrenia and Bipolar Disorder. *Neuropharmacology* **2017**, *112*, 297–306. https://doi.org/10.1016/j.neuropharm.2016.05.020.
7. Hunt, C.; Macedo, E. Cordeiro, T.; Suchting, R.; de Dios, C.; Cuellar Leal, V.A.; Soares, J.C.; Dantzer, R.; Teixeira, A.L.; Selvaraj, S. Effect of Immune Activation on the Kynurenine Pathway and Depression Symptoms—A Systematic Review and Meta-Analysis. *Neurosci. Biobehav. Rev.* **2020**, *118*, 514–523. https://doi.org/10.1016/j.neubiorev.2020.08.010.
8. Brown, S.J.; Huang, X.-F.; Newell, K.A. The Kynurenine Pathway in Major Depression: What We Know and Where to Next. *Neurosci. Biobehav. Rev.* **2021**, *127*, 917–927. https://doi.org/10.1016/j.neubiorev.2021.05.018.
9. Serafini, G.; Adavastro, G.; Canepa, G.; Capobianco, L.; Conigliaro, C.; Pittaluga, F.; Murri, M.B.; Valchera, A.; De Berardis, D.; Pompili, M.; et al. Abnormalities in Kynurenine Pathway Metabolism in Treatment-Resistant Depression and Suicidality: A Systematic Review. *CNS Neurol. Disord. Drug Targets* **2017**, *16*, 440–453. https://doi.org/10.2174/1871527316666170413110605.
10. Cao, B.; Chen, Y.; Ren, Z.; Pan, Z.; McIntyre, R.S.; Wang, D. Dysregulation of Kynurenine Pathway and Potential Dynamic Changes of Kynurenine in Schizophrenia: A Systematic Review and Meta-Analysis. *Neurosci. Biobehav. Rev.* **2021**, *123*, 203–214. https://doi.org/10.1016/j.neubiorev.2021.01.018.
11. Tan, V.X.; Guillemin, G.J. Kynurenine Pathway Metabolites as Biomarkers for Amyotrophic Lateral Sclerosis. *Front. Neurosci.* **2019**, *13*, 1013. https://doi.org/10.3389/fnins.2019.01013.
12. Pukoli, D.; Polyák, H.; Rajda, C.; Vécsei, L. Kynurenines and Neurofilament Light Chain in Multiple Sclerosis. *Front. Neurosci.* **2021**, *15*, 658202. https://doi.org/10.3389/fnins.2021.658202.
13. Sharma, V.K.; Singh, T.G.; Prabhakar, N.K.; Mannan, A. Kynurenine Metabolism and Alzheimer's Disease: The Potential Targets and Approaches. *Neurochem. Res.* **2022**, *47*, 1459–1476. https://doi.org/10.1007/s11064-022-03546-8.
14. Liang, Y.; Xie, S.; He, Y.; Xu, M.; Qiao, X.; Zhu, Y.; Wu, W. Kynurenine Pathway Metabolites as Biomarkers in Alzheimer's Disease. *Dis. Markers* **2022**, *2022*, e9484217. https://doi.org/10.1155/2022/9484217.
15. Tanaka, M.; Bohár, Z.; Vécsei, L. Are Kynurenines Accomplices or Principal Villains in Dementia? Maintenance of Kynurenine Metabolism. *Molecules* **2020**, *25*, 564. https://doi.org/10.3390/molecules25030564.

16. Venkatesan, D.; Iyer, M.; Narayanasamy, A.; Siva, K.; Vellingiri, B. Kynurenine Pathway in Parkinson's Disease-an Update. *Eneurologicalsci* **2020**, *21*, 100270. https://doi.org/10.1016/j.ensci.2020.100270.
17. Behl, T.; Kaur, I.; Sehgal, A.; Singh, S.; Bhatia, S.; Al-Harrasi, A.; Zengin, G.; Bumbu, A.G.; Andronie-Cioara, F.L.; Nechifor, A.C.; et al. The Footprint of Kynurenine Pathway in Neurodegeneration: Janus-Faced Role in Parkinson's Disorder and Therapeutic Implications. *Int. J. Mol. Sci.* **2021**, *22*, 6737. https://doi.org/10.3390/ijms22136737.
18. Mor, A.; Tankiewicz-Kwedlo, A.; Krupa, A.; Pawlak, D. Role of Kynurenine Pathway in Oxidative Stress during Neurodegenerative Disorders. *Cells* **2021**, *10*, 1603. https://doi.org/10.3390/cells10071603.
19. Török, N.; Tanaka, M.; Vécsei, L. Searching for Peripheral Biomarkers in Neurodegenerative Diseases: The Tryptophan-Kynurenine Metabolic Pathway. *Int. J. Mol. Sci.* **2020**, *21*, 9338. https://doi.org/10.3390/ijms21249338.
20. Huang, Y.-S.; Ogbechi, J.; Clanchy, F.I.; Williams, R.O.; Stone, T.W. IDO and Kynurenine Metabolites in Peripheral and CNS Disorders. *Front. Immunol.* **2020**, *11*, 388. https://doi.org/10.3389/fimmu.2020.00388.
21. Curto, M.; Lionetto, L.; Fazio, F.; Mitsikostas, D.-D.; Martelletti, P. Fathoming the Kynurenine Pathway in Migraine: Why Understanding the Enzymatic Cascades Is Still Critically Important. *Intern. Emerg. Med.* **2015**, *10*, 413–421. https://doi.org/10.1007/s11739-015-1208-6.
22. Fila, M.; Chojnacki, J.; Pawlowska, E.; Szczepanska, J.; Chojnacki, C.; Blasiak, J. Kynurenine Pathway of Tryptophan Metabolism in Migraine and Functional Gastrointestinal Disorders. *Int. J. Mol. Sci.* **2021**, *22*, 10134. https://doi.org/10.3390/ijms221810134.
23. Martos, D.; Tuka, B.; Tanaka, M.; Vécsei, L.; Telegdy, G. Memory Enhancement with Kynurenic Acid and Its Mechanisms in Neurotransmission. *Biomedicines* **2022**, *10*, 849. https://doi.org/10.3390/biomedicines10040849.
24. Tanaka, M.; Vécsei, L. Monitoring the Kynurenine System: Concentrations, Ratios or What Else? *Adv. Clin. Exp. Med.* **2021**, *30*, 775–778. https://doi.org/10.17219/acem/139572.
25. Dogrul, B.N. Indolamine 2,3-Dioxygenase (IDO) Inhibitors as a Potential Treatment for Somatic Symptoms. *Med. Hypotheses* **2022**, *160*, 110777. https://doi.org/10.1016/j.mehy.2022.110777.
26. Tanaka, M.; Török, N.; Tóth, F.; Szabó, Á.; Vécsei, L. Co-Players in Chronic Pain: Neuroinflammation and the Tryptophan-Kynurenine Metabolic Pathway. *Biomedicines* **2021**, *9*, 897. https://doi.org/10.3390/biomedicines9080897.
27. Ciapała, K.; Mika, J.; Rojewska, E. The Kynurenine Pathway as a Potential Target for Neuropathic Pain Therapy Design: From Basic Research to Clinical Perspectives. *Int. J. Mol. Sci.* **2021**, *22*, 11055. https://doi.org/10.3390/ijms222011055.
28. Jovanovic, F.; Candido, K.D.; Knezevic, N.N. The Role of the Kynurenine Signaling Pathway in Different Chronic Pain Conditions and Potential Use of Therapeutic Agents. *Int. J. Mol. Sci.* **2020**, *21*, 6045. https://doi.org/10.3390/ijms21176045.
29. Heng, B.; Lim, C.K.; Lovejoy, D.B.; Bessede, A.; Gluch, L.; Guillemin, G.J. Understanding the Role of the Kynurenine Pathway in Human Breast Cancer Immunobiology. *Oncotarget* **2016**, *7*, 6506–6520. https://doi.org/10.18632/oncotarget.6467.
30. Kiluk, M.; Lewkowicz, J.; Pawlak, D.; Tankiewicz-Kwedlo, A. Crosstalk between Tryptophan Metabolism via Kynurenine Pathway and Carbohydrate Metabolism in the Context of Cardio-Metabolic Risk-Review. *J. Clin. Med.* **2021**, *10*, 2484. https://doi.org/10.3390/jcm10112484.

31. Song, P.; Ramprasath, T.; Wang, H.; Zou, M.-H. Abnormal Kynurenine Pathway of Tryptophan Catabolism in Cardiovascular Diseases. *Cell. Mol. Life Sci.* **2017**, *74*, 2899–2916. https://doi.org/10.1007/s00018-017-2504-2.
32. Gáspár, R.; Halmi, D.; Demján, V.; Berkecz, R.; Pipicz, M.; Csont, T. Kynurenine Pathway Metabolites as Potential Clinical Biomarkers in Coronary Artery Disease. *Front. Immunol.* **2021**, *12*, 768560. https://doi.org/10.3389/fimmu.2021.768560.
33. Razquin, C.; Ruiz-Canela, M.; Toledo, E.; Hernández-Alonso, P.; Clish, C.B.; Guasch-Ferré, M.; Li, J.; Wittenbecher, C.; Dennis, C.; Alonso-Gómez, A.; et al. Metabolomics of the Tryptophan-Kynurenine Degradation Pathway and Risk of Atrial Fibrillation and Heart Failure: Potential Modification Effect of Mediterranean Diet. *Am. J. Clin. Nutr.* **2021**, *114*, 1646–1654. https://doi.org/10.1093/ajcn/nqab238.
34. Melhem, N.J.; Taleb, S. Tryptophan: From Diet to Cardiovascular Diseases. *Int. J. Mol. Sci.* **2021**, *22*, 9904. https://doi.org/10.3390/ijms22189904.
35. Engin, A.B.; Engin, A. The Interactions Between Kynurenine, Folate, Methionine and Pteridine Pathways in Obesity. In *Obesity and Lipotoxicity*; Engin, A.B., Engin, A., Eds.; Advances in Experimental Medicine and Biology; Springer International Publishing: Cham, Switzerland, 2017; pp. 511–527, ISBN 978-3-319-48382-5.
36. Dadvar, S.; Ferreira, D.M.S.; Cervenka, I.; Ruas, J.L. The Weight of Nutrients: Kynurenine Metabolites in Obesity and Exercise. *J. Intern. Med.* **2018**, *284*, 519–533. https://doi.org/10.1111/joim.12830.
37. Agus, A.; Planchais, J.; Sokol, H. Gut Microbiota Regulation of Tryptophan Metabolism in Health and Disease. *Cell Host Microbe* **2018**, *23*, 716–724. https://doi.org/10.1016/j.chom.2018.05.003.
38. Kennedy, P.J.; Cryan, J.F.; Dinan, T.G.; Clarke, G. Kynurenine Pathway Metabolism and the Microbiota-Gut-Brain Axis. *Neuropharmacology* **2017**, *112*, 399–412. https://doi.org/10.1016/j.neuropharm.2016.07.002.
39. Sun, M.; Ma, N.; He, T.; Johnston, L.J.; Ma, X. Tryptophan (Trp) Modulates Gut Homeostasis via Aryl Hydrocarbon Receptor (AhR). *Crit. Rev. Food Sci. Nutr.* **2020**, *60*, 1760–1768. https://doi.org/10.1080/10408398.2019.1598334.
40. Chen, L.-M.; Bao, C.-H.; Wu, Y.; Liang, S.-H.; Wang, D.; Wu, L.-Y.; Huang, Y.; Liu, H.-R.; Wu, H.-G. Tryptophan-Kynurenine Metabolism: A Link between the Gut and Brain for Depression in Inflammatory Bowel Disease. *J. Neuroinflamm.* **2021**, *18*, 135. https://doi.org/10.1186/s12974-021-02175-2.
41. Krupa, A.; Kowalska, I. The Kynurenine Pathway-New Linkage between Innate and Adaptive Immunity in Autoimmune Endocrinopathies. *Int. J. Mol. Sci.* **2021**, *22*, 9879. https://doi.org/10.3390/ijms22189879.
42. Haq, S.; Grondin, J.A.; Khan, W.I. Tryptophan-Derived Serotonin-Kynurenine Balance in Immune Activation and Intestinal Inflammation. *FASEB J.* **2021**, *35*, e21888. https://doi.org/10.1096/fj.202100702R.
43. Moffett, J.R.; Arun, P.; Puthillathu, N.; Vengilote, R.; Ives, J.A.; Badawy, A.A.-B.; Namboodiri, A.M. Quinolinate as a Marker for Kynurenine Metabolite Formation and the Unresolved Question of NAD+ Synthesis During Inflammation and Infection. *Front. Immunol.* **2020**, *11*, 31. https://doi.org/10.3389/fimmu.2020.00031.
44. Collier, M.E.; Zhang, S.; Scrutton, N.S.; Giorgini, F. Inflammation Control and Improvement of Cognitive Function in COVID-19 Infections: Is There a Role for Kynurenine 3-Monooxygenase Inhibition? *Drug Discov. Today* **2021**, *26*, 1473–1481. https://doi.org/10.1016/j.drudis.2021.02.009.
45. Lawler, N.G.; Gray, N.; Kimhofer, T.; Boughton, B.; Gay, M.; Yang, R.; Morillon, A.-C.; Chin, S.-T.; Ryan, M.; Begum, S.; et al. Systemic Perturbations in Amine and Kynurenine Metabolism Associated with

Acute SARS-CoV-2 Infection and Inflammatory Cytokine Responses. *J. Proteome Res.* **2021**, *20*, 2796–2811. https://doi.org/10.1021/acs.jproteome.1c00052.

46. Wee, H.N.; Liu, J.-J.; Ching, J.; Kovalik, J.-P.; Lim, S.C. The Kynurenine Pathway in Acute Kidney Injury and Chronic Kidney Disease. *Am. J. Nephrol.* **2021**, *52*, 771–787. https://doi.org/10.1159/000519811.
47. Mor, A.; Kalaska, B.; Pawlak, D. Kynurenine Pathway in Chronic Kidney Disease: What's Old, What's New, and What's Next? *Int. J. Tryptophan Res. IJTR* **2020**, *13*, 1178646920954882. https://doi.org/10.1177/1178646920954882.
48. Krishnamurthy, S.; Gilot, D.; Ahn, S.B.; Lam, V.; Shin, J.-S.; Guillemin, G.J.; Heng, B. Involvement of Kynurenine Pathway in Hepatocellular Carcinoma. *Cancers* **2021**, *13*, 5180. https://doi.org/10.3390/cancers13205180.
49. Miyazaki, T.; Chung, S.; Sakai, H.; Ohata, H.; Obata, Y.; Shiokawa, D.; Mizoguchi, Y.; Kubo, T.; Ichikawa, H.; Taniguchi, H.; et al. Stemness and Immune Evasion Conferred by the TDO2-AHR Pathway Are Associated with Liver Metastasis of Colon Cancer. *Cancer Sci.* **2022**, *113*, 170–181. https://doi.org/10.1111/cas.15182.
50. Martin, K.S.; Azzolini, M.; Lira Ruas, J. The Kynurenine Connection: How Exercise Shifts Muscle Tryptophan Metabolism and Affects Energy Homeostasis, the Immune System, and the Brain. *Am. J. Physiol. Cell Physiol.* **2020**, *318*, C818–C830. https://doi.org/10.1152/ajpcell.00580.2019.
51. Sui, G.; Jia, L.; Quan, D.; Zhao, N.; Yang, G. Activation of the Gut Microbiota-Kynurenine-Liver Axis Contributes to the Development of Nonalcoholic Hepatic Steatosis in Nondiabetic Adults. *Aging* **2021**, *13*, 21309–21324. https://doi.org/10.18632/aging.203460.
52. Dhillon, A.K.; Rupp, C.; Bergquist, A.; Voitl, R.; Folseraas, T.; Trøseid, M.; Midttun, Ø.; Ueland, P.M.; Karlsen, T.H.; Vesterhus, M.; et al. Associations of Neopterin and Kynurenine–Tryptophan Ratio with Survival in Primary Sclerosing Cholangitis. *Scand. J. Gastroenterol.* **2021**, *56*, 443–452. https://doi.org/10.1080/00365521.2021.1880627.
53. Clària, J.; Moreau, R.; Fenaille, F.; Amorós, A.; Junot, C.; Gronbaek, H.; Coenraad, M.J.; Pruvost, A.; Ghettas, A.; Chu-Van, E.; et al. Orchestration of Tryptophan-Kynurenine Pathway, Acute Decompensation, and Acute-on-Chronic Liver Failure in Cirrhosis. *Hepatology* **2019**, *69*, 1686–1701. https://doi.org/10.1002/hep.30363.
54. Kardashian, A.; Ma, Y.; Yin, M.T.; Scherzer, R.; Nolan, O.; Aweeka, F.; Tien, P.C.; Price, J.C. High Kynurenine:Tryptophan Ratio Is Associated With Liver Fibrosis in HIV-Monoinfected and HIV/Hepatitis C Virus–Coinfected Women. *Open Forum Infect. Dis.* **2019**, *6*, ofz281. https://doi.org/10.1093/ofid/ofz281.
55. Ogbechi, J.; Clanchy, F.I.; Huang, Y.-S.; Topping, L.M.; Stone, T.W.; Williams, R.O. IDO Activation, Inflammation and Musculoskeletal Disease. *Exp. Gerontol.* **2020**, *131*, 110820. https://doi.org/10.1016/j.exger.2019.110820.
56. Vyavahare, S.; Kumar, S.; Cantu, N.; Kolhe, R.; Bollag, W.B.; McGee-Lawrence, M.E.; Hill, W.D.; Hamrick, M.W.; Isales, C.M.; Fulzele, S. Tryptophan-Kynurenine Pathway in COVID-19-Dependent Musculoskeletal Pathology: A Minireview. *Mediators Inflamm.* **2021**, *2021*, 2911578. https://doi.org/10.1155/2021/2911578.
57. Broekhuizen, M.; Danser, A.H.J.; Reiss, I.K.M.; Merkus, D. The Function of the Kynurenine Pathway in the Placenta: A Novel Pharmacotherapeutic Target? *Int. J. Environ. Res. Public. Health* **2021**, *18*, 11545. https://doi.org/10.3390/ijerph182111545.

58. Silvano, A.; Seravalli, V.; Strambi, N.; Cecchi, M.; Tartarotti, E.; Parenti, A.; Di Tommaso, M. Tryptophan Metabolism and Immune Regulation in the Human Placenta. *J. Reprod. Immunol.* **2021**, *147*, 103361. https://doi.org/10.1016/j.jri.2021.103361.
59. Badawy, A.A.-B. Tryptophan Metabolism, Disposition and Utilization in Pregnancy. *Biosci. Rep.* **2015**, *35*, e00261. https://doi.org/10.1042/BSR20150197.
60. Xu, K.; Liu, G.; Fu, C. The Tryptophan Pathway Targeting Antioxidant Capacity in the Placenta. *Oxid. Med. Cell. Longev.* **2018**, *2018*, 1054797. https://doi.org/10.1155/2018/1054797.
61. Worton, S.A.; Greenwood, S.L.; Wareing, M.; Heazell, A.E.; Myers, J. The Kynurenine Pathway; A New Target for Treating Maternal Features of Preeclampsia? *Placenta* **2019**, *84*, 44–49. https://doi.org/10.1016/j.placenta.2019.04.007.
62. Gumusoglu, S.; Scroggins, S.; Vignato, J.; Santillan, D.; Santillan, M. The Serotonin-Immune Axis in Preeclampsia. *Curr. Hypertens. Rep.* **2021**, *23*, 37. https://doi.org/10.1007/s11906-021-01155-4.
63. Ala, M. The Footprint of Kynurenine Pathway in Every Cancer: A New Target for Chemotherapy. *Eur. J. Pharmacol.* **2021**, *896*, 173921. https://doi.org/10.1016/j.ejphar.2021.173921.
64. Castro-Portuguez, R.; Sutphin, G.L. Kynurenine Pathway, NAD+ Synthesis, and Mitochondrial Function: Targeting Tryptophan Metabolism to Promote Longevity and Healthspan. *Exp. Gerontol.* **2020**, *132*, 110841. https://doi.org/10.1016/j.exger.2020.110841.
65. Savitz, J. The Kynurenine Pathway: A Finger in Every Pie. *Mol. Psychiatry* **2020**, *25*, 131–147. https://doi.org/10.1038/s41380-019-0414-4.
66. Badawy, A.A.-B. Kynurenine Pathway and Human Systems. *Exp. Gerontol.* **2020**, *129*, 110770. https://doi.org/10.1016/j.exger.2019.110770.
67. Lu, Y.; Shao, M.; Wu, T. Kynurenine-3-Monooxygenase: A New Direction for the Treatment in Different Diseases. *Food Sci. Nutr.* **2020**, *8*, 711–719. https://doi.org/10.1002/fsn3.1418.
